# Improved outcome in hip fracture patients in the aging population following co-managed care compared to conventional surgical treatment: a retrospective, dual-center cohort study

**DOI:** 10.1186/s12877-019-1289-6

**Published:** 2019-11-27

**Authors:** Carl Neuerburg, Stefan Förch, Johannes Gleich, Wolfgang Böcker, Markus Gosch, Christian Kammerlander, Edgar Mayr

**Affiliations:** 1 Department of General, Trauma and Reconstructive Surgery, University Hospital Ludwig-Maximilians-University (LMU) Munich, Marchioninistr. 15, D-81377 Munich, Germany; 2 Department of Trauma, Orthopedic, Hand and Reconstructive surgery, University Hospital Augsburg, Augsburg, Germany; 3 Department of Medicine 2/Geriatrics, General Hospital Nuremberg, Paracelsus Medical University, Nuremberg, Germany

**Keywords:** Orthogeriatrics, Hip fracture, Integrated care, Frailty

## Abstract

**Background:**

Hip fracture patients in the aging population frequently present with various comorbidities, whilst preservation of independency and activities of daily living can be challenging. Thus, an interdisciplinary orthogeriatric treatment of these patients has recognized a growing acceptance in the last years. As there is still limited data on the impact of this approach, the present study aimed to evaluate the long-term outcome in elderly hip fracture patients, by comparing the treatment of a hospital with integrated orthogeriatric care (OGC) with a conventional trauma care (CTC).

**Methods:**

We conducted a retrospective, two-center, cohort study. In two maximum care hospitals all patients presenting with a hip fracture at the age of ≥ 70 years were consecutively assigned within a 1 year period and underwent follow-up examination 12 months after surgery. Patients treated in hospital site A were treated with an interdisciplinary orthogeriatric approach (co-managed care), patients treated in hospital B underwent conventional trauma care. Main outcome parameters were 1 year mortality, readmission rate, requirement of care (RC) and personal activities of daily living (ADL).

**Results:**

A total of 436 patients were included (219 with OGC / 217 with CTC). The mean age was 83.55 (66–99) years for OGC and 83.50 (70–103) years for CTC (76.7 and 75.6% of the patients respectively were female). One year mortality rates were 22.8% (OGC) and 28.1% (CTC; *p* = 0.029), readmission rates were 25.7% for OGC compared to 39.7% for CTC (*p* = 0.014). Inconsistent data were found for activities of daily living. After 1 year, 7.8% (OGC) and 13.8% (CTC) of the patients were lost to follow-up.

**Conclusions:**

Interdisciplinary orthogeriatric management revealed encouraging impact on the long-term outcome of hip fracture patients in the aging population. The observed reduction of mortality, requirements of care and readmission rates to hospital clearly support the health-economic impact of an interdisciplinary orthogeriatric care on specialized wards.

**Trial registration:**

The study was approved and registered by the bavarian medical council (BLAEK: 7/11192) and the local ethics committee of munich university (Reg. No. 234–16) and was conducted as a two-center, cohort study at a hospital with integrated orthogeriatric care and a hospital with conventional trauma care.

## Background

Older hip fracture patients are often characterized by various comorbidities and geriatric syndromes, such as sarcopenia, which can induce frailty [1]. In these patients, preservation of independency and activities of daily living is of superior importance and it is the goal of treatment to avoid a further functional decline [2, 3]. There is an estimated increase in the annual amount of hip fracture patients up to 6.3 mn / year by 2050 [4]. This trend is related to the demographic changes of our ageing population, in Germany i.e. the amount of people at the age > 70 years is expected to double until 2050 [5], which will be a huge burden for health-care systems. Besides improved surgical care, the 1 year mortality rate among hip fracture patients is reported to be as high as 25–30% [6, 7]. To broadly address the complex needs of older trauma patients, special treatment models have been developed, merging the expertise of geriatricians and orthopaedic surgeons in different ways [8]. Out of these models, full integration of a geriatrician in the team of orthopaedic surgeons is expected to be the most effective approach for interdisciplinary treatment [9] and was often established within the last years [8]. Impact of the geriatrician can already start preoperatively i.e. with an individual risk assessment of each patient, for example with the Nottingham Hip Fracture score [10]. Depending on the individual risk factors potential preoperative optimization of the patients’ general health can be adapted i.e. via intravascular volume restoration, pain and medication management. Especially the postoperative course is significantly influenced by the geriatrician with prevention of delirium, reduction of polypharmacy and identification of inappropriate medication such as management of multimorbidity in frail patients. However, given the limited amount of randomized controlled trials, present investigations scarcely observed significant benefits attributed to the interdisciplinary management of older trauma patients [11]. The majority of recent studies investigating the impact of orthogeriatric care (OGC) were retrospective studies, using data before and after the implementation of OGC [12–14]. They demonstrated improved 30-day mortality as well as 1 year mortality. Nevertheless, this study design could bring BIAS because of a learning process during implementation, while reliable results can only be obtained after a longer period of time [15]. A prospective, randomized controlled trial conducted by Prestmo et al. showed positive effects on mobility, activities of daily living and cognition following OGC [16]. Other present investigations chose strict in−/exclusion criteria such as exclusion of cognitive disordered patients, which might not reflect the typical orthogeriatric population.

Therefore, the aim of this study was to evaluate the impact of OGC in a comparative two-center approach in which patients were assigned and analyzed at one-year follow-up with regards to 1 year mortality, hospital readmission and requirements of care (RC) in comparison to conventional trauma care (CTC).

## Methods

The study was approved and registered by the bavarian medical council (BLAEK: 7/11192) and the local ethics committee of munich university (Reg. No. 234–16) and was conducted as a retrospective, two-center cohort study at a hospital with integrated orthogeriatric care and a hospital with conventional trauma care.

### Study center structures

Both are level one trauma centers and the trauma units have a capacity of 123 beds in hospital site A (including 44 OGC beds) and 93 (CTC) beds in site B.

The OGC unit at hospital site A was implemented in 2008. All trauma patients admitted to this unit are aged > 70 years and treated following specific geriatric assessment. For specific geriatric risk assessment in the emergency department geriatric screening according to Lachs et al. was used [17]. The patients underwent care according to the previously described model by Pioli et al. [8]. All elements of orthogeriatric care (according to Lisk et al. [18]) were considered, including daily interdisciplinary rounds and activating care by specialized nurses and physiotherapists. Each patient received two sessions of physiotherapy per day (2 × 30 minutes), while parts of this were performed as group therapy (Table 1). Primary objective was the earliest possible mobilization out of bed as an attempt to regain patients’ independency. To prevent or treat postoperative delirium, a clearly structured daily schedule was given, which starts with activating body care with assistance in the morning, followed by shared breakfast with other patients (if possible) and the first physiotherapy session, then lunch and second session and ends with supper. Also regular interdisciplinary team meetings including surgeons, geriatricians, nurses, physiotherapists, social workers and others addressed the individual patients’ needs.

**Table 1:** Structure and treatment at study centers

	CTC	OTC
Department	• Department of Trauma Surgery • Other departments on consultation basis	• Department of Trauma Surgery with geriatricians working within the team
Facilities	• Trauma ward: Single-triple bed rooms on different trauma wards with up to 30 beds	• Specific designed orthogeriatric ward: Single-double-bed rooms on one ward with up to 44 beds
Treatment	• Early mobilization after surgery • Physiotherapy 1x/day (30 mins) • Social care workers on call	• Early mobilization after surgery • Physiotherapy 2x/day (30 mins) • “activating care”: help for body care with greatest possible participation of the patient, shared meals with other patients in a common room with independent transfer (as possible) • Interdisciplinary treatment with focus on: Somatic health, mental health, function and social situation

At hospital site B patients were treated on a conventional trauma ward at the time of the study. Treatment was generally managed by trauma surgeons and their team, who had no specific geriatric expertise. Geriatric assessment was not performed during inpatient treatment. Other departments were consulted in case of need, but there was no permanent multidisciplinary treatment approach. Surgical treatment was performed or supervised by specialists according to the principles of the AO Foundation at both hospital sites.

### Patient recruitment

All hip fracture patients aged ≥ 70 years presenting to the study centers from 01/01/2014 to 31/12/2014 were consecutively included. Patients with ASA score 4 or 5 were excluded because of pre-existing critical illness and therefore high risk of dying regardless the kind of care they receive. There were no other inclusion or exclusion criteria to get a realistic depiction of patients population. Both hospitals take care for a large city including surrounding area and are only 70 km away from each other, so homogeneous distribution of each economic and ethnic status should be given. Baseline data were collected and the two groups were checked for comparability by distribution of age, sex, type of fracture and treatment, time to surgery, ASA (American Society of Anaesthesiologists) score and discharge destination.

### Outcome parameters

One year mortality rate was defined as primary outcome. Secondary outcomes were the readmission rate, requirement of care, place of residence, personal activities of daily living (ADL) measured with the Barthel Index, length of stay and the patients’ perspective of their status.

### Data collection and management

A standardized data management file was used at both hospital sites and merged blinded following completion of follow-up (Excel 2011, Version 14.0 for Mac OS X, Microsoft Cooperation, Redmond). Baseline data were collected from the medical records.

Twelve months after their stay in hospital, each patient was contacted by phone or was sent a specifically designed questionnaire asking for the variables listed above by two of the authors. If the patient could not answer by himself, their next of kin or caregiver was asked for consent and information. In case that no information was available via contact of the patients themselves, relatives, general practitioners, nursing homes or the local authorities, the patient was regarded as „lost to follow-up“. We cross-checked all lost to follow-up patients with the hospital data management system at the 1 year follow up, so in case of readmission more information about their current status and the reason of readmission were obtained.

### Assessment instruments

Table 2 shows the degrees of RC according to the German health-care assurance, which was extended up to five degrees in January 2017. A degree can be requested for each patient, independent from place of residence (own home or nursing home). It is determined as the total of time a patient needs professional help in his activities of daily living and is assessed by specially qualified physicians of the insurance companies. The degree before admission and 1 year after hospitalization was requested.

**Table 2:** Requirement of care according to the German health-care assurance

Degree	Care needed per day
0	patient is independent in his activities of daily living or needs minimal support
1	at least 90 min per day
2	at least 180 min per day
3	patient needs care 24 h/day

Readmission rate was assessed by retrospective analysis of the data management system of each study center and also queried by the patients themselves (to register also readmission to a non-study center hospital). Reasons for readmission were separated into complications associated with surgery, re-fracture and other medical complications.

The place of residence was assessed at admission and at 1 year of follow-up, divided in sheltered housing (independent living in a specialized living community with professional help only when required), nursing home (living in a specialized facility with professional care by nurses 24 h per day and 7 days a week, possible for a short period of time until return to home or on a long-term basis) and own home.

For personal activities of daily living, the Barthel Index (BI) [19] was assessed as a reliable score for patient-reported outcome measures (range from 0 to 100, higher score suggests higher independence in daily living [20]). CTC had no regular geriatric assessment at the time of the study. Therefore, only at OGC BI was assessed at the time of hospitalization, at discharge and by the time of follow-up examination.

To asses the treatment success from the patients’ perspective, the current status of health was queried compared to the status of health before the fracture. Therefore, we designed a scale with 5 qualities to choose from much worse to worse, unchanged, better, to much better. Regarding to the study population this assessment should be well understandable and as simple as possible, so we forewent to use more complex questionnaires.

### Statistics

IBM SPSS Statistics for Macintosh, Version 24 (IBM Corp. Released 2016. Amonk, NY) was used for statistical analysis. We report normally distributed continuous variables as means with ranges, categorical data as absolute frequency with a percentage distribution.

Single imputation using the expectation maximization algorithm was used for isolated missing items on the questionnaires after checking them for MCAR (missing completely at random). Scores from the same date were used as predictors.

We used the chi-square test and the Fisher exact test to identify a possible relationship between categorical variables and also to compare the 1 year mortality rates between OGC and CTC. Mann-Whitney test, Kolmogorov-Smirnov test and t-test were used depending on data distribution. A *p*-value < 0.05 was regarded to be statistically significant.

We followed the instructions of the STROBE panel to arrange this manuscript.

## Results

**Fig. 1:**
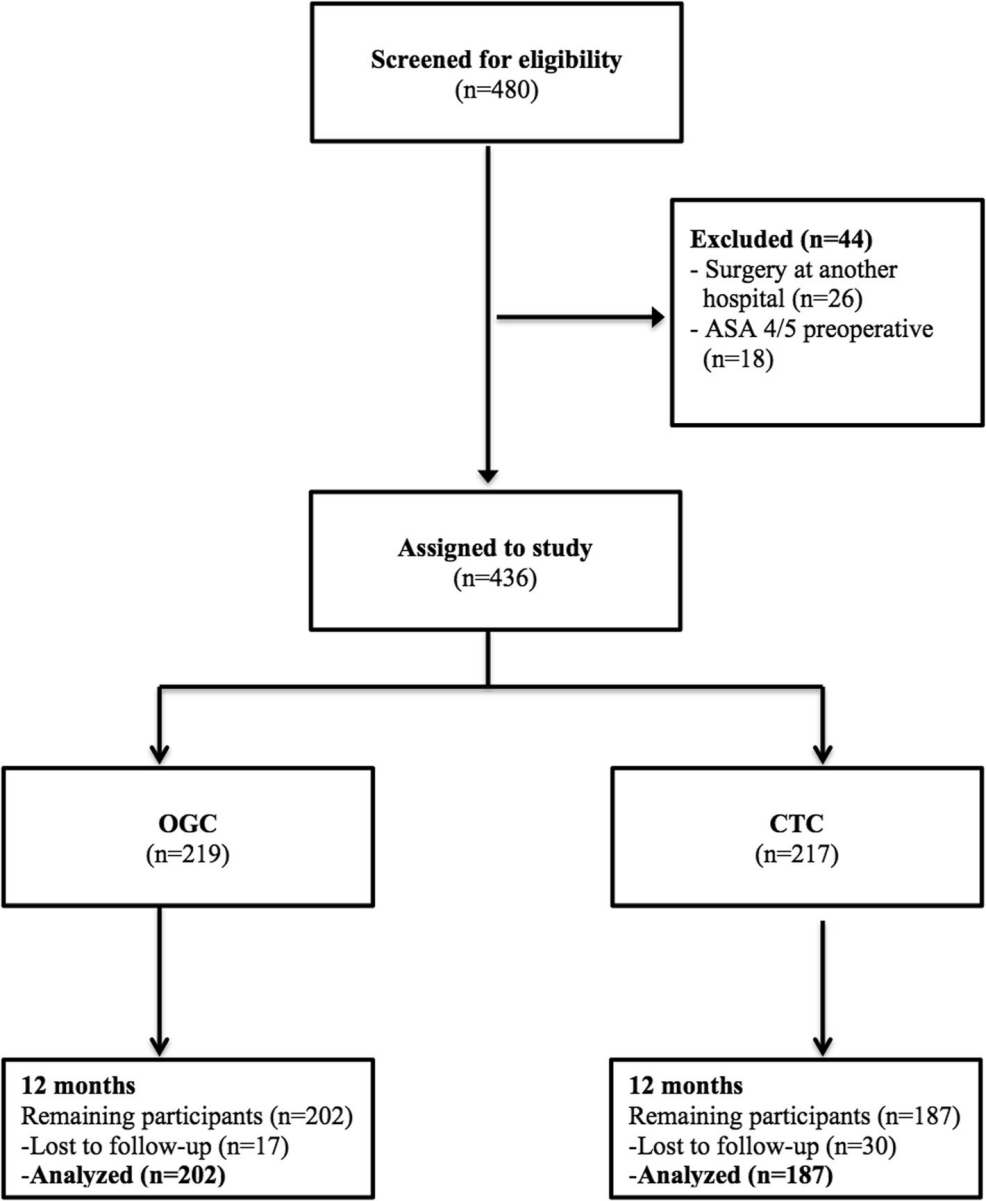
Flowchart of patients who met inclusion criteria for the study

A total of 480 patients were screened for eligibility, 231 following OGC and 249 after CTC. Twelve patients at OGC and 32 patients at CTC were excluded as they underwent surgery in another hospital and were transferred thereafter to one of the study hospitals or as they presented preoperative with an ASA score of 4 or 5 and therefore were in critical condition, regardless of the type of treatment (Fig. 1; Legend: “Flowchart of patients who met inclusion criteria for the study”). Table 3 shows the baseline patient characteristics as absolute numbers with percentage distribution written in parentheses such as mean values and additional standard deviation.

**Table 3:** Baseline data

	CTC	OGC	*p*-value
Age (range)	83.50(70–103)	83.55(69–99)	0.943
Gender			0.823
Female	164(75.6%)	168(76.7%)	
Male	53(24.4%)	51(23.3%)	
ASA			0.063
1	3(1.4%)	5(2.3%)	
2	104(47.9%)	81(37.0%)	
3	110(50.7%)	133(60.7%)	
length of stay (range)	13.51(1–31)	17.67(1–134)	< 0.001
Barthel Index (range)	only at follow-up	31.72(0–90)	
Prefracture living			0.021
at home	188(86.6%)	169(77.2%)	
nursing home	29(13.4%)	48(21.9%)	
sheltered housing	0(0%)	2(0.9%)	
Discharged to			< 0.001
own home	32(14.7%)	18(8.2%)	
post-acute care	128(59.0%)	147(67.1%)	
short-term care	13(6.0%)	13(5.9%)	
nursing home	20(9.2%)	35(16.0%)	
other	24(11.1%)	6(2.7%)	
Fracture type			
trochanteric (total)	**110(50.7%)**	**105(47.9%)**	0.123
AO type A1	38(34.5%)	24(22.9%)	
AO type A2	53(48.2%)	64(61.0%)	
AO type A3	19(17.3%)	17(16.2%)	
femoral neck (total)	**93(42.9%)**	**92(42.0%)**	0.015
Garden I	14(15.1%)	2(2.2%)	
Garden II	24(25.8%)	24(26.1%)	
Garden III	28(30.1%)	38(41.3%)	
Garden IV	27(29.0)	28(30.4%)	
periprosthetic (total)	7(3.2%)	13(5.9%)	0.734
other (total)	7(3.2%)	9(4.1%)	
Surgical treatment trochanteric			0.143
conservative	1(0.9%)	0(0.0%)	
arthroplasty	3(2.8%)	0(0.0%)	
nailing / screw	106(96.4%)	105(100.0%)	
Surgical treatment femoral neck fractures			< 0.001
conservative	3(3.2%)	0(0.0%)	
arthroplasty	60(64.5%)	92(100.0%)	
nailing / screw	30(32.3%)	0(0.0%)	
Surgical treatment periprosthetic fractures/others			
conservative	5(35.7%)	0(0.0%)	
nailing / screw	9(64.3%)	13(59.1%)	
other	0(0.0%)	9(40.9%)	
time to surgery			0.912
< 24 h	151(72.6%)	158(72.1%)	
> 24 h	57(26.3%)	61(27.9%)	
lost to follow up	30(13.8%)	17(7.8%)	

After 1 year, 7.8% (*n* = 17) following OGC and 13.8% (*n* = 30) of the patients undergoing CTC were lost to follow-up. The reasons for that were refusal by the patient / its proxy, moving to a new home without information about the new address, no permanent residence, residence in a foreign country and non availability either in writing or by phone.

In the group of patients lost to follow-up no differences were found at the time of discharge with regards to age (*p* = 0.094), gender (0.782), ASA score (0.104), present diagnosis of dementia (0.932), prefracture type of living (0.31) and length of stay (0.074), so we assume that outcome should not be influenced by the missing patients.

In-hospital mortality was 1.8% (*n* = 4) for CTC and 2.7% (*n* = 6) for OGC, with these patients included, one year mortality was found to be 28.1% (*n* = 61) for CTC and significantly lower in patients being treated via OGC, 22.8% (*n* = 50; *p* = 0.029). The number needed to treat was 12.71 with a relative risk reduction of 24.1% for OGC.

Readmission rates in the first year following hip fracture surgery were significantly reduced in patients undergoing OGC with 25.7% (*n* = 39) compared to 39.7% (*n* = 50) of CTC patients (*p* = 0.014).

Reason for readmission was due to medical reasons independent from initial surgery in most of the cases, including 76.9% for OGC (*n* = 30) such as 52.0% of the CTC patients (*n* = 26). Readmission due to surgical reasons appeared in 10.3% (*n* = 4) of OGC patients and 20.0% (*n* = 10) of the CTC patients. Secondary fracture occurred in 12.8% (*n* = 5) of the OGC patients and 28.0% (*n* = 14) of the CTC patients.

Requirement of care was significantly higher for OGC patients in all subgroups at the time of admission (*p* < 0.001). At that time, 51.9% (*n* = 56) of the OGC patients had no RC (degree 0), compared to 81.6% (*n* = 102) of the CTC patients. 34.3% (*n* = 37) of the patients treated in the OGC department were admitted with RC degree 1, 13.9% (*n* = 15) with degree 2 and 0% (*n* = 0) of the patients had an RC degree of 3. In the department with CTC, 12.8% (*n* = 16) of the patients were observed with a RC degree 1 by the time of admission, 3.2% (*n* = 4) with degree 2 and 2.4% (*n* = 3) of the patients with RC degree 3.

After 1 year, significantly more of the patients initially graded degree 1 were raised to a higher RC degree following conventional trauma care (68.8%, *n* = 11) compared to patients that underwent surgery in the OGC department (29.7%, *n* = 11; *p* = 0.014). No significant differences were found in patients raised from RC degree 0 to a higher RC degree at the department of CTC compared to OGC (25.5% (*n* = 26) vs. 35.7% (*n* = 20); *p* = 0.202). Also no significant differences were observed in patients with RC degree 2 at the time of hip fracture surgery which were raised to grade 3 by the time of follow-up examination (CTC 0%(*n* = 0) vs. OGC 13.3% (*n* = 2); *p* = 0.614). RC degree 3 is not stated, as there is no increase possible.

Evaluation of the place of residence after 1 year revealed distinct differences between OGC and CTC treated patients in all subgroups (Table 4).

**Table 4:** Place of residence at one-year follow-up compared to the time of admission to hospital

Place of residence at the time of hip fracture	CTC	OGC	*p*-value
own home	-return (87.9%, n = 102) -nursing home (9.5%, *n* = 11) -sheltered housing (2.6%, *n* = 3)	-return (72.5%, *n* = 79) -nursing home (20.2%, *n* = 22) -sheltered housing (7.3%, *n* = 8)	0.013
nursing home	-return to home (0%, *n* = 0) -return to nh (55.6%, *n* = 5) -sheltered housing (44.4%, *n* = 4)	-return to home (20.7%, *n* = 6) -return to nh (75.9%, *n* = 22) -sheltered housing (3.4%, *n* = 1)	0.004
sheltered housing	−0%(*n* = 0)	-return to home (100%, *n* = 2)	

Generally more patients in the CTC group who were living at their own homes at the time of hip fracture were still living there at one-year follow-up compared to OGC treated patients. Significantly more of the patients treated with OGC were transferred to care facilities as observed for patients treated with CTC (*p* = 0.013).

**Fig. 2:**
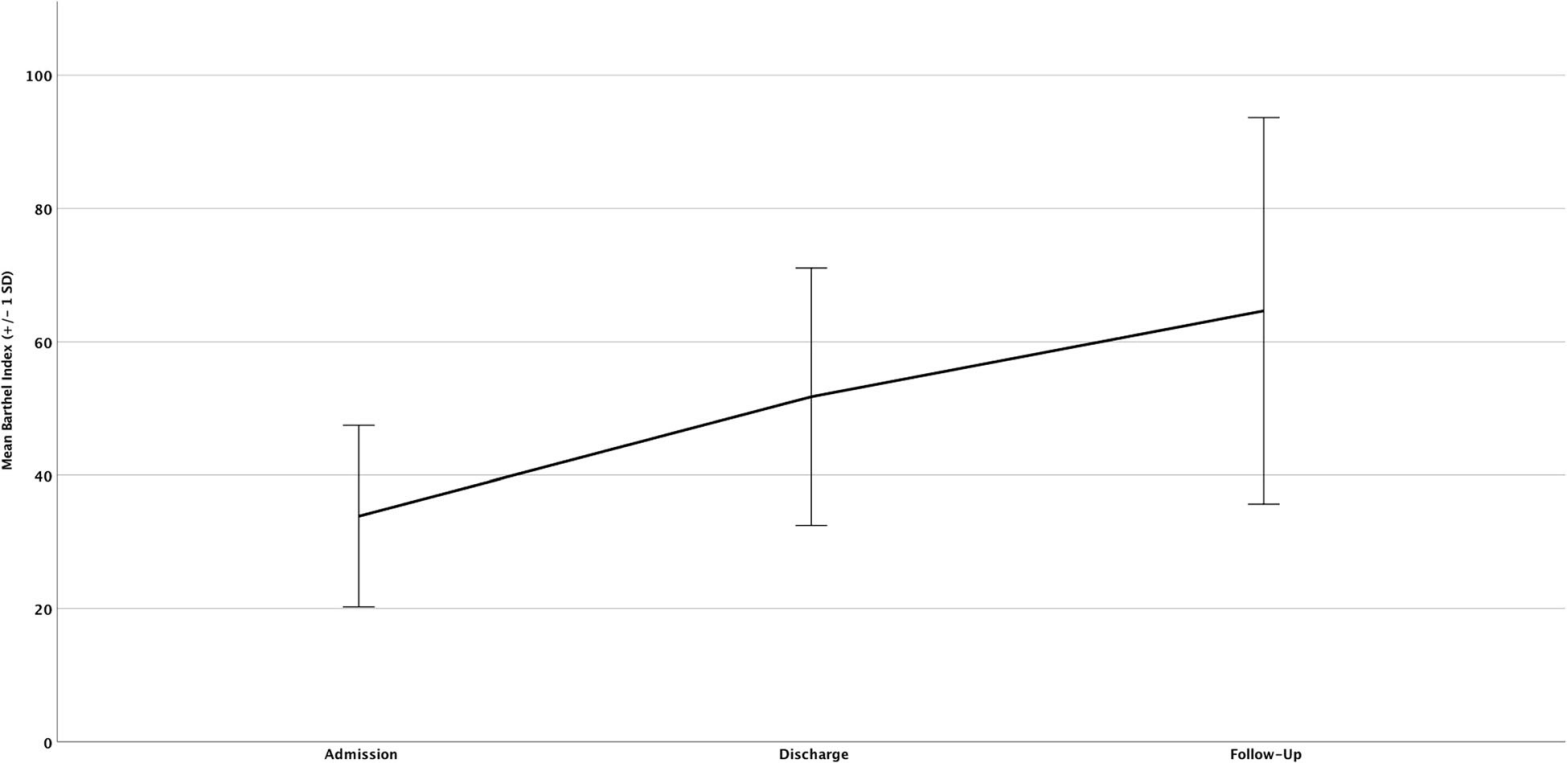
Mean Barthel Index at admission, discharge and follow-up + standard deviation (SD) for OGC patients

At admission to OGC, mean Barthel Index was 31.72 (range 0–90), at discharge 45.93 (range 0–90) and at 1 year follow up 64.68 (range 0–100), indicating a significant increase of the BI following hip fracture surgery in the first year (*p* < 0.001, Fig. 2; Legend: “Mean Barthel Index at admission, discharge and follow-up for OGC patients”). Also a paired t-test was performed to investigate changes in pre- and post-interventional Barthel Index following impact of OGC. Results of 108 matched pairs show significant improvements, with a discharge BI of 51,76 (SD 19,302) and a follow-up BI of 64,68 (SD 29,012) (*p* < 0.001).

In patients being treated with CTC there was no standardized geriatric assessment at that time, so the BI at admission and discharge was scarcely available. Only the mean BI at follow-up investigation was recorded, which was 73.88 (range 0–100). There was a significant difference (*p* = 0.024) at 1 year follow up compared to the BI of the OGC group, but this could only be considered as a snapshot because of the missing admission and discharge values.

**Fig. 3:**
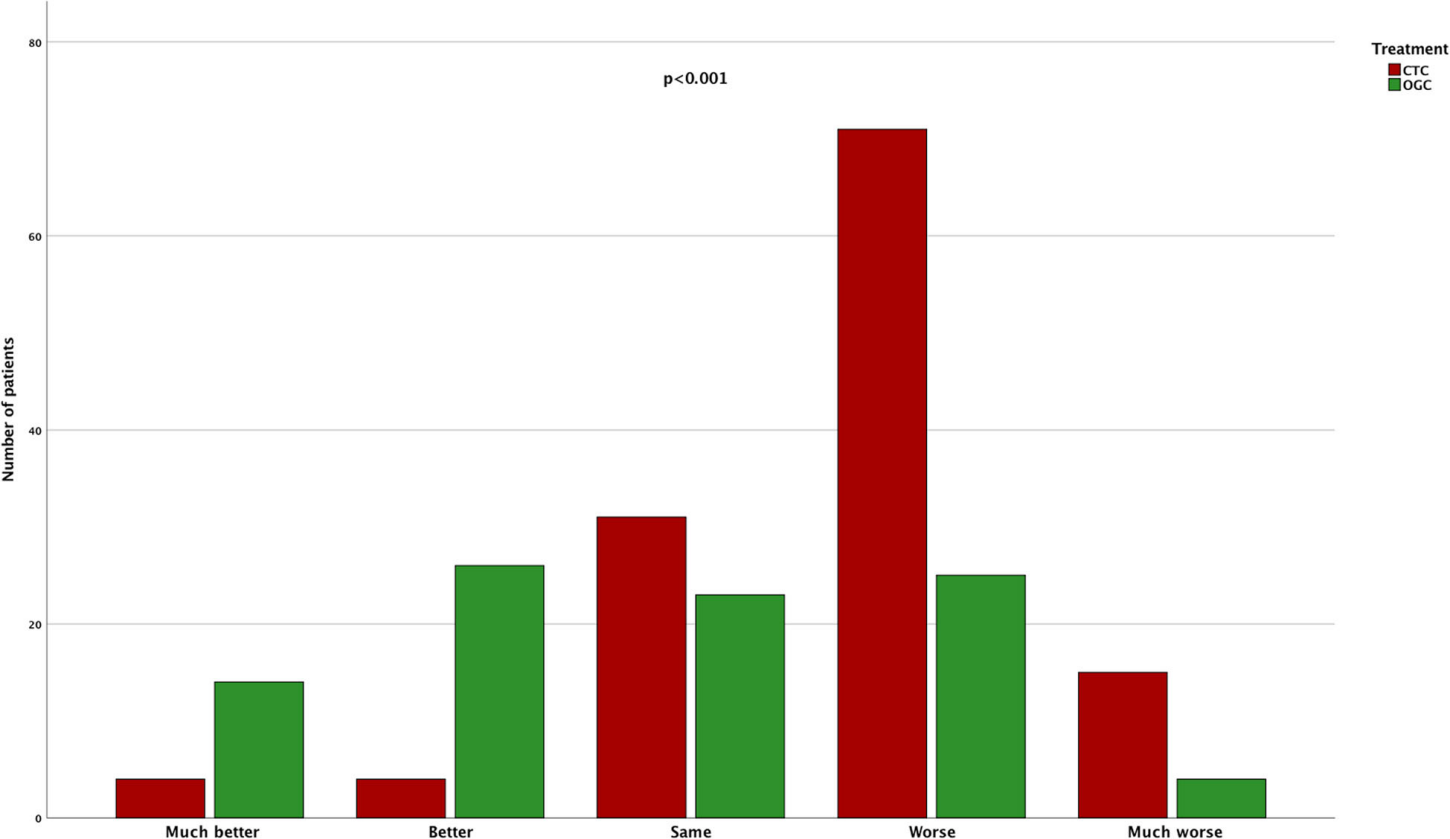
Status of health queried with a 5 qualities questionnaire at the time of follow-up examination 12 month after hip fracture surgery compared to the status of health before the fracture in both OGC and CTC treated patients

Assessment of the patients condition at 1 year follow up compared to the condition before admission to hospital revealed, that significantly (*p* < 0.001) more patients in the OGC group state a better status of health than in the CTC group (Fig. 3; Legend: “Status of health 12 month after hip fracture surgery compared to status before the fracture. Queried with a 5 qualities questionnaire in both OGC and CTC treated patients“).

## Discussion

Interdisciplinary treatment approaches experience a growing acceptance for the treatment of geriatric trauma patients in order to preserve activities of daily living and independency. Although implementation of a successful orthogeriatric co-managed model of care varies from one hospital to another, some key elements have to be considered. According to Lisk et al. the key elements of orthogeriatric care are: prompt admission to orthopaedic care; rapid and comprehensive medical, surgical and anaesthesiologic assessment; minimal delay to surgery; accurate and well-performed surgery (single-shot surgery); prompt mobilization and rehabilitation; early supported discharge and ongoing community rehabilitation; secondary fracture prevention [18]. Considering the additional resources needed for a comprehensive orthogeriatric care model, the impact of this interdisciplinary approach is frequently discussed and data remain controversial [16, 21]. Therefore, the present study aimed to evaluate the key differences in the short and long-term at 1 year follow-up in a comparative study design on hip fracture patients treated with different models of care at two trauma centers of maximum care. In this comparative study three major differences were observed with regards to the long-term outcome following hip fracture surgery in an interdisciplinary approach of OGC.

At first, one-year mortality was significantly reduced in patients being treated in the OGC unit compared to CTC. These results are in accordance with existing investigations on the impact of OGC and appear to be one of the major benefits attributed to OGC [12, 14, 22–24]. Only 5 years ago comparative trials on the impact of orthogeriatic treatment on long-term mortality stated, that no significant differences were found in between the groups, while the number of comparative trials taken into account at that time was limited with relatively small study groups [11]. A more recent meta-analysis conducted by Moyet et al. showed that patients with hip fracture in the aging population admitted early into any sort of orthogeriatric models or more specifically to a dedicated orthogeriatric ward had reduced long-term mortality, while the authors also claimed, that randomised controlled trials on that topic are still missing [24]. Therefore, similar to hip replacement registries, there is a growing need for registries focusing trauma patients in the aging population such as the orthogeriatric trauma registry founded by the german trauma society (AltersTraumaRegister-DGU®).

There are various impacts known to affect mortality such as a reduced time to index surgery, intensive postoperative mobilization, limited time of bed rest and rapid treatment of perioperative complications such as urinary tract infections, pneumonia and others [25]. One significant difference that could have influenced mortality as well was the difference in the surgical approach in femoral neck fractures. Although more non-displaced femoral neck fractures were observed in the CTC group, osteosynthesis i.e. with cannulated screw fixation was performed to a significantly greater extend in CTC treated patients compared to the surgical techniques observed in the orthogeriatric trauma center that favoured hip replacement (Table 3). There is growing acceptance that in terms of a single-shot surgery, hip replacement is superior for the treatment of even minimally displaced femoral neck fractures, as there is a remaining failure following fracture fixation in femoral neck fractures of up to 22% [26, 27]. However, as complications arising from initial surgery, such as posttraumatic arthritis, avascular necrosis of the hip and others generally lead to secondary surgery in the long run, the present study with a follow-up period of 12 month might be missing subsequent surgical interventions in the group of CTC treated patients.

Also physiotherapy exercises were more intense in the OGC treated patients compared to CTC patients, which might have had an influence on the patients’ fall risk and mortality. Rapp K et al. conducted an analysis on orthogeriatric patients admitted to sub-acute care units in a geriatric rehabilitation clinic and stated, that the overall fall rate was 10.2 falls/1000 person-days with highest fall risks during the first week and decreasing risks within the following weeks [28]. Therefore, the use of a risk assessment tool for osteoporotic fracture prevention could be of additional value for further comparative trials to assess impact of orthogeriatric programs. Given the present study, it could not be identified which aspect of OGC affected the observed 1 year-mortality the most.

Secondly, a significant reduction of readmission rates was observed for patients being treated in the OGC department, which supports the theory, that patients were discharged to an optimized domestic environment compared to CTC patients.

Also the significantly prolonged hospital stay in OGC treated patients could have affected the patient’s discharge status. Interdisciplinary co-managed care of multimorbid patients aims to improve the patients’ status of health prior to discharge and can be associated with a prolonged hospital stay. Up to now there is only little evidence on the impact of the length of stay in relation to long-term outcome. Nikkel et al. stated, that a prolonged inpatient stay is associated with a higher mortality rate, yet the authors investigated only the short term period of 30 days in a retrospective and rather heterogenous study design, that also included very young patients > 50 years [29]. In frail orthogeriatric patients it remains questionable, if early discharge is beneficial for the individuals’ outcome and should be the basis for further investigations.

Furthermore, it was shown that significantly more patients undergoing OGC were transferred to a care facility and these patients were able to maintain their requirement of care (RC). However, these data have to be interpreted with caution, as there were already more patients with a preexisting RC in the group of OGC patients compared to CTC treated patients. Data on the requirement and distribution of nursing facilities in older trauma patients are generally rare and there are only few investigations on that topic. There have been reports on variations in nursing home discharge rates for urban and rural nursing facility residents with hip fracture [30]. Therefore, the observed differences in the requirement of care facilities may also be attributed to rural differences in between the investigated study centers. Nevertheless, within a 1 year period in the group of CTC treated patients a significant increase in the degree of care was observed, which was associated with more frequent changes from the initial degree of care to a higher level of care. These findings indicate a greater loss of independency in the postoperative course within the group of patients being treated in the CTC department, which highlights the importance of an individualized management of discharge. As stated above the supported discharge and ongoing community rehabilitation remains a key element of orthogeriatric care. Thus, one could assume, that the involvement of geriatricians and the close interaction to the social workers in the department with OGC may be associated with a more individualized adjustment of the patient’s needs. However, considering the retrospective study design, missing information in the patients that had passed away could have biased the findings on requirements of care and need to be taken into account.

Thirdly, with regards to the activities of daily living, inconsistent findings were observed. While the Barthel Index at one-year follow-up was significantly higher for CTC patients, the assessment of the patients’ condition revealed that significantly more of the OGC treated patients state a better status of health after 12 month following hip fracture surgery. As stated above, the difference in BI could only be considered as a snapshot. It has already been shown, that co-management by geriatricians and orthopedic surgeons with a combined standardized care, leads to improved processes and outcomes for patients with hip fractures in the short term [30]. It appears reasonable, that the effects on activities of daily living might decline in the long-term following orthogeriatric treatment. Yet, another study on orthogeriatric care by Doshi HK et al. reported a significant functional improvement, which was still observed at 1 year follow-up [32]. Encouraging results were also found by some of our authors in another study where they could reveal, that orthogeriatric co-management improves the outcome especially in long-term care residents with fragility fractures [33]. Other investigations stated, that previous walking ability and the presence of complications, such as pressure ulcers or delirium, play a greater role in functional recovery than cognitive impairment, which again supports the treatment with an interdisciplinary approach [34]. Thus, implementation of a continuous rehabilitation program following hip fracture surgery should be given special consideration in future as an attempt to improve secondary fracture prevention.

## Limitations

Although the assessment of patients’ comorbidities was of particular importance during the study period and comorbidities were recorded with great diligence, the fact that no geriatric assessment was performed in the department of CTC might have led to a loss of some comorbidities, which could have affected the outcome. Also the BI recorded in the department of CTC at time of the study period was not collected homogenously, which is why we refrained from evaluating the BI in the CTC department during this period. However, it has been shown by Mayoral AP et al. recently, that the BI is a reliable tool for assessment of activity in osteoporotic hip fracture patients while an increase of the BI in the first year can be observed [35]. As the BI at one-year follow-up was higher for CTC patients we believe, that patient groups were also comparable by the time of admission to hospital, which is supported by demographic aspects regarding age, sex and ASA score which displayed a homogeneity of the study groups. To our believe, the present study therefore achieved to give a comparable and realistic view on older hip fracture patients population and deficiencies of their treatment at two level one trauma centers which is also a strength of the present study, hazarding the consequences of a possible bias by broad inclusion of patients. We also believe, that the BI is only one important parameter for follow up evaluation in these patients.

Furthermore, as the investigated departments of trauma surgery are located in different areas, patients were transferred to different clinics of rehabilitation following in-patient treatment for hip fracture surgery. While similar programs of rehabilitation are provided by the patients insurance, differences in activities of daily living at one-year follow-up might also be affected by minor differences in the rehabilitation programs of the individual rehabilitation unit. Besides that, in a retrospective study on older hip fracture patients using health insurance claims data from Germany, it was shown that a lack of inpatient rehabilitation was significantly associated with a worsening care level [36].

## Conclusions

To the best of our knowledge, the present study is the first two-center study evaluating the impact of OGC by comparing two level one trauma centers. Taken together, the results strongly support the concept of an interdisciplinary orthogeriatric approach for the treatment of hip fracture patients in the aging population. Considering the findings listed above dedicated perioperative hip fracture co-management programs have shown to be cost-effective in high-volume centers [37]. While the health care system in the United Kingdom has considered the impact of an interdisciplinary treatment of hip fractures in the aging population for a while given the “Best practice tariff”, the topic is gaining a growing relevance at present. In Germany a report of health care providers was published just recently in which 221 hospitals were identified which do not meet the necessary requirements for the treatment of hip fractures in the aging population, while treatment of these patients is prospectively reserved for specialized centers [38].

As a consequence of the study, a fully integrated geriatrician has been recruited in the present CTC department by now and adjustments of the local circumstances were made. Since June 2016 the initial department of CTC is now registered as a certified orthogeriatric trauma center by the German Society of Trauma Surgery (DGU).

## Data Availability

The datasets generated and/or analysed during the current study are not publicly available as data was pseudonymised, but are available from the corresponding author on reasonable request.

## Abbreviations

ADL: Activities of daily living
AO Foundation: Arbeitsgemeinschaft für Osteosynthesefragen
ASA: American Society of Anaesthesiologists
BI: Barthel index
CTC: Conventional trauma care
DGU: German Society of Trauma Surgery
MCAR: Missing completely at random
OGC: Orthogeriatric care
RC: Requirement of care
